# Evaluating the Assembly Dynamics in the Human Vaginal Microbiomes With Niche-Neutral Hybrid Modeling

**DOI:** 10.3389/fmicb.2021.699939

**Published:** 2021-08-20

**Authors:** Zhanshan (Sam) Ma

**Affiliations:** ^1^Computational Biology and Medical Ecology Lab, State Key Laboratory of Genetic Resources and Evolution, Kunming Institute of Zoology, Chinese Academy of Sciences, Kunming, China; ^2^Center for Excellence in Animal Evolution and Genetics, Chinese Academy of Sciences, Kunming, China

**Keywords:** unified neutral theory of biodiversity and biogeography, multi-site neutral model, niche-Neutral hybrid model, human vaginal microbiome, bacterial vaginosis, hierarchical Dirichlet process

## Abstract

Using 2,733 longitudinal vaginal microbiome samples (representing local microbial communities) from 79 individuals (representing meta-communities) in the states of healthy, BV (bacterial vaginosis) and pregnancy, we assess and interpret the relative importance of stochastic forces (e.g., stochastic drifts in bacteria demography, and stochastic dispersal) vs. deterministic selection (e.g., host genome, and host physiology) in shaping the dynamics of human vaginal microbiome (HVM) diversity by an integrated analysis with multi-site neutral (MSN) and niche-neutral hybrid (NNH) modeling. It was found that, when the traditional “default” *P*-value = 0.05 was specified, the neutral drifts were predominant (≥50% metacommunities indistinguishable from the MSN prediction), while the niche differentiations were moderate (<20% from the NNH prediction). The study also analyzed two challenging uncertainties in testing the neutral and/or niche-neutral hybrid models, i.e., lack of full model specificity – non-unique fittings of same datasets to multiple models with potentially different mechanistic assumptions – and lack of definite rules for setting the *P*-value thresholds (also noted as *P*_*t*_-value when referring to the threshold of *P*-value in this article) in testing null hypothesis (model). Indeed, the two uncertainties can be interdependent, which further complicates the statistical inferences. To deal with the uncertainties, the MSN/NNH test results under a series of *P*-values ranged from 0.05 to 0.95 were presented. Furthermore, the influence of *P*-value threshold-setting on the model specificity, and the effects of woman’s health status on the neutrality level of HVM were examined. It was found that with the increase of *P*-value threshold from 0.05 to 0.95, the overlap (non-unique) fitting of MSN and NNH decreased from 29.1 to 1.3%, whereas the specificity (uniquely fitted to data) of MSN model was kept between 55.7 and 82.3%. Also with the rising *P*-value threshold, the difference between healthy and BV groups become significant. These findings suggested that traditional single *P*-value threshold (such as the *de facto* standard *P-*value = 0.05) might be insufficient for testing the neutral and/or niche neutral hybrid models.

## Introduction

The relationship between human vaginal microbiome (HVM) and women’s health has been investigated since the 1980s, when clinical microbiologists had postulated that the diversity and possibly stability of vaginal microbiome are involved in the occurrence/recurrence of bacterial vaginosis (BV) (e.g., Sobel, 1999; Fredricks et al., 2005; Fredricks, 2011; Ma et al., 2012). Those studies are among the earliest ecological approaches to diseases now often referred to as the human MADs (microbiome-associated diseases) with an ever more rapidly growing list including BV, IBD (inflammatory bowel disease), periodontitis, cystic fibrosis (CF), psoriasis and many others (Lynch and Pedersen, 2016; Knight et al., 2017; Young, 2017; Gilbert et al., 2018). The metagenomics technique and the launch of the human microbiome project (HMP) and MetaHIT (metagenome of human intestinal tract) have revolutionized the investigation of the human microbiome and associated diseases during the last decade or so. Nevertheless, many questions in the field are still open and new more complex questions are being raised. In the case of BV and vaginal microbiome, as described by Fredricks (2011) who borrowed Winston Churchill’s words for a very different topic, “*BV remains a riddle, wrapped in a mystery, and inside an enigma*.” A recent characterization “*that BV is not a single entity, but a syndrome linked to various community types that cause somewhat similar physiological symptoms*.” by Ma et al. (2012) reflects the state-of-the-art understanding of BV etiology. Obviously, although the importance of vaginal microbiome ecology in BV etiology is repeatedly confirmed, the mechanistic relationship between BV and HVM is far from clear. A pair of questions of fundamental importance: what are the underlying mechanisms driving the dynamics of HVM and what are their implications to the occurrence/recurrence of BV, are still largely unanswered.

Addressing the question of community assembly and diversity maintenance, the essential ingredients of community structure and dynamics, has attracted extensive attention and also led to vigorous debate (Alonso et al., 2006; McGill et al., 2006; Chisholm and Pacala, 2010; Rosindell et al., 2011). Two leading and competing theories in this field have been the traditional niche theory with a history back to the 1910s (Grinnell, 1917; Hutchinson, 1957; Holt, 2009) and more recent neutral theory (Hubbell, 2001). Both theories were invented to explain a familiar phenomenon on the earth, which was described by Darwin (1859) in the last paragraph of his “*On the Origin of Species*” as “*It is interesting to contemplate a tangled bank, clothed with many plants of many kinds, with birds singing on the bushes, with various insects flitting about, and with worms crawling through the damp earth, and to reflect that these elaborately constructed forms, so different from each other, and dependent upon each other in so complex a manner, have all been produced by laws acting around us.”* In modern ecological terminology, *entangled bank* is essentially the concept of ecological *community*. Darwin was wondering how diverse lives (species) could coexist and form a beautiful entangled bank, while his theory stipulated the universal struggle for life as a consequence to natural selection. The classic niche theory assumed that each species has its own niche in which its individuals are adapted to live and prosper, and the entangled bank consists of many different niches suitable for many different species. In terms of niche theory, deterministic traits a species possess or selective niche forces play critical roles in driving the assembly of an ecological community as well as the maintenance of diversity after the community is established.

In the late 1990s, Hubbell (2001) challenged the traditional niche view by proposing the unified neutral theory of biodiversity and biogeography (UNTB). Different from traditional niche theory, the UNTB was formulated as a probability distribution model, which can be fitted with the species abundance distribution data (the number of each species in a community), obtainable by sampling ecological communities, and rigorously tested statistically. The theory assumes that the individuals of all species in a community are demographically equivalent, but their birth/death rates are stochastic, which means birth-death, migration, and speciation are all random events. Consequently, random drift and dispersal play critical roles in driving community assembly and diversity maintenance. Some researchers argued that the concept of species equivalence is “flawed” given the existence of niche differences and competitive asymmetries among species. Nevertheless, the stochasticity in species demography (particularly of single-cell microbes) is also a biological reality and its role may not be ignored in many communities. In reality, both deterministic niche forces and stochastic neutral forces may be in effect in setting the rules of community assembly and diversity maintenance, and it may be the *hybrid* effects that shape the community dynamics. For this reason, in the last decade and so, several hybrid models that integrate neutral and niche effects have been developed (e.g., Tilman, 2004; Ofiteru et al., 2010; Stokes and Archer, 2010; Jeraldo et al., 2012; Pigolotti and Cencini, 2013; Tang and Zhou, 2013; Fisher and Mehta, 2014; Kalyuzhny et al., 2014a, b, 2015; Matthews and Whittaker, 2014; Noble and Fagan, 2015). As to the debates on the usefulness and validity of the UNTB, using an analogy, in modern statistics (especially in biostatistics), it has been widely recognized that many datasets do not follow the Gaussian distribution (the normal distribution); nevertheless, few statisticians would question the foundational role of the Gaussian distribution, not to mention its validity. Similarly, the merits and unique advantage of UNTB as a null model for testing the significance of stochastic drift and dispersal have been firmly established and widely applied in the community ecology of plants and animals.

In the present study, we use a pair of models, the first a multi-site neutral model (Harris et al., 2017) and the second, a niche-neutral hybrid model (Tang and Zhou, 2013), to evaluate the relative significance of neutral and niche effects in shaping the dynamics of HVM. We further investigate the difference in the neutral-niche *continuum* between BV patients and healthy women. Our approach is different from most existing applications of neutral or hybrid models in the following three aspects.

First, most existing neutral or niche-neutral hybrid models use spatially implicit/explicit community/metacommunity data, whereas we use longitudinal (time-series) sampling of the community/metacommunity. In spatially explicit models, the metacommunity consists of multiple local communities, which are connected with each other through dispersal and migration. In temporal (time-series) models, the metacommunity consists of a series of “snapshots” of the same community at different time points, i.e., the time-series data obtained from sampling the vaginal microbiome of a subject at different time points in this study. Indeed, previously, Kalyuzhny et al. (2014a, b, 2015) used time-series data to perform dynamic analysis of the niches versus neutrality and they termed the analysis as a generalized neutral theory for explaining the static and dynamic properties of ecological communities. A reason we did not adopt their models is that the models we use in this study, as explained below, are truly multi-site mechanistically, which are mapped to the time-series points in our study.

Second, we use a *truly* multi-site neutral (MSN) model of UNTB, which was developed by Harris et al. (2017) to overcome the severe computational limitation of existing neutral theory models when the number of local communities is large and the migration rates among the local communities are different (Etienne, 2007, 2009a,b). The core technique Harris et al. (2017) developed was to approximate the multi-site UNTB model with the hierarchical Dirichlet process (HDP) and use an efficient Bayesian machine-learning algorithm. With their approach, fitting even the largest dataset can be performed in a reasonable amount of time. This important computational advance enables us to build a UNTB model for each subject by utilizing the time series sampling of her vaginal microbiome. This capability is of significant practical importance given the established connection between BV and the diversity of the vaginal microbiome, in particular, a long-standing puzzle in BV etiology – the rise of species diversity associated with BV (e.g., Sobel, 1999; Fredricks et al., 2005; Fredricks, 2011; Ma et al., 2012; Ma and Ellison, 2018, 2019).

Third, we also apply the niche-neutral hybrid (NNH) model by Tang and Zhou (2013) to further assess the neutral-niche hybrid effects in shaping the dynamics of HVM diversity. A major reason we prefer this hybrid model to other existing hybrid models (e.g., Tilman, 2004; Ofiteru et al., 2010; Stokes and Archer, 2010; Jeraldo et al., 2012; Pigolotti and Cencini, 2013; Tang and Zhou, 2013; Fisher and Mehta, 2014; Kalyuzhny et al., 2014a, b, 2015; Matthews and Whittaker, 2014; Noble and Fagan, 2015) is because both MSN and NNH use exactly the same data collection methods – either multi-site or multi-time-point sampling. The only essential difference between the neutral-niche hybrid model (NNH) and multi-site neutral model (MSN) is the assumption that niche differences exist among local communities in NNH, while the MSN assumes no niche differentiation. In a time-series setting, the NNH model can tell us whether deterministic forces (similar to habitat selection in a spatial setting) such as when changes in the host’s physiology significantly influence the dynamics of vaginal microbiome diversity over time.

**FIGURE 1:**
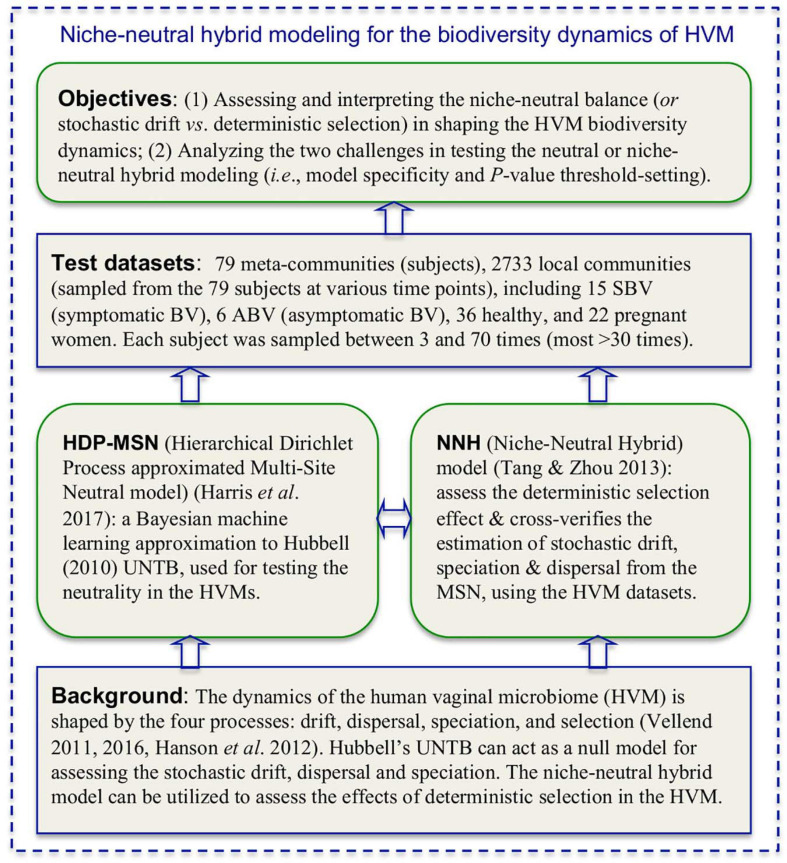
A diagram illustrating the background, objectives and the integrated niche-neutral theoretic-approach to achieving the objectives of this study based on 2,733 longitudinal metagenomics (16S-rRNA) samples collected from 79 women (including BV patients).

In summary, by building and testing the MSN and NNH models for each subject, we are able to evaluate the relative importance of stochastic forces (e.g., neutral dispersal, drift, and stochastic diversification) vs. deterministic forces (e.g., microbial interactions, host genome and physiology, menses, etc.) in shaping the dynamics of community diversity. Furthermore, if we treat BV or health status as part of the host physiology, testing the MSN/NNH models can reveal the impact of BV on the dynamics of the HVM diversity (assuming that diversity change is the consequence of BV), or reveal the diversity changes that induce BV (assuming that diversity change is the cause of BV). Regardless of the causal assumption, our approach offers a useful tool for evaluating the mechanisms (niche vs. neutral) of the dynamics of HVM diversity as well as the factors affecting the balances between different mechanisms. We demonstrate our approach (see Figure 1) by using the datasets (see Table 1) from three separate longitudinal studies on the HVM, including 79 subjects sampled at 2,733 time points (Gajer et al., 2012; Ravel et al., 2013; Romero et al., 2014).

**TABLE 1:** The datasets of multi-site HVM (human vaginal microbiome) datasets utilized for testing the MSN (multi-site neutral) and NNH (niche-neutral hybrid) models.

Datasets	*N	**S	Sample description	Sources
ABV (Asymptomatic Bacterial Vaginosis)	6	66∼70	Ravel et al. (2013) sampled and DNA-pyrosequenced the vaginal microbiota of a cohort of 25 subjects over a 10-week period, consisting of 15 SBV, 6 ABV, and 4 healthy subjects (HEA-1). Total 16S-rRNA reads = 8,757,681, Average reads = 5285, The dataset is available from: 10.1186/2049-2618-1-2	Ravel et al. (2013) *Microbiome*
SBV (Symptomatic Bacterial Vaginosis)	15	59∼70		
HEA-1 (Healthy 1)	4	66∼69		
HEA-2 (Healthy 2): “32-healthy” cohort of HVMC study	32	25∼33	Gajer et al. (2012) sampled and DNA-pyrosequenced the vaginal microbiota of a cohort of 32 healthy individuals (HEA-2). Total 16S-rRNA reads = 2,522,080 from 937 samples; Average reads = 2692. The OTU table is available at: 10.1126/scitranslmed.3003605	Gajer et al. (2012) *Science Translational Medicine*
PREG (Pregnancy)	22	3∼8	The vaginal microbiomes of a cohort of 22 normally pregnant women were sampled 6 times (for each individual) and DNA pyrosequenced. Total 16S-rRNA reads = 567,448; Average reads = 4082; The dataset is available at: https://doi.org/10.1186/2049-2618-2-4	Romero et al. (2014) *Microbiome*
Total or range	79	3∼70	A total of 79 meta-communities and 2,733 local communities (time-series samples) were sampled to conduct the tests.	

*N = the number of subjects (individuals) included in each dataset. **S = the approximate number of *time points* when samples of the HVMC were taken from each individual.

## Materials and Methods

### Human Vaginal Microbiome (HVM) Datasets and Analysis Strategy

Table 1 below listed the three published datasets (groups) of the HVM (human vaginal microbiome), which are reanalyzed in this study to perform the niche-neutral theoretic analysis. Figure 1 is a diagram illustrating the background, objectives and the integrated niche-neutral approach to achieving the objectives of this study outlined previously. Two mathematical models: the multi-site neutral model (MSN) by Harris et al. (2017) and niche-neutral hybrid model (NNH) by Tang and Zhou (2013), are used to fit the same HVM datasets. Both the models are extensions or derived from Hubbell (2001) unified neutral theory of biodiversity and biogeography (UNTB). Brief description of the MSN and NNH models as well as their fittings is presented in Table 1. It is noted that these datasets are from three independent studies: minor difference in sequencing protocols may exist. For this reason, the samples from each of the 79 individuals are modeled independently. With the independent modeling, the influence from sequencing protocols should have minimized.

### The Multi-Site Neutral Model (MSN) by Harris et al. (2017)

#### Hubbell’s Unified Neutral Theory of Biodiversity (UNTB)

The UNTB conceptually distinguishes between metacommunity dynamics from local community dynamics coupled to metacommunity through migrations. The theory assumes that both the dynamics are driven by similar neutral processes, except that in metacommunity speciation, rather than migration are in operations (Hubbell, 2001, 2006). The neutral process or ecological equivalence between species implies that the demographic rates (birth/death) of all species are stochastic but equivalent on per capita basis (Harris et al., 2017). There are three key parameters (elements) with the UNTB, the immigration rate (*I*_*i*_), which controls the coupling of a local community to the metacommunity. Another is the speciation rate, also known as the fundamental biodiversity number (θ), which can be interpreted as the rate at which new individuals are added to the metacommunity due to speciation. The third aspect of the UNTB is to assume that the SAD (species abundance distribution) of each community sample can be described by the multinomial (MN) distribution, formally:

(1) $$\bar{X_{i}}∼M\,N\,\left(N_{i},\,{\bar{\pi }}_{i}\right)$$

where *N*_*i*_ is the size of *i*-th local community, ${\bar{\pi }}_{i}$ is a vector of the probability of observing a particularly species at *i*-th local community (Harris et al., 2017).

#### UNTB-HDP (Hierarchical Dirichlet Process) Limit to Metacommunities

A fully general case of fitting multiple sites (local communities) UNTB with potentially different immigration rates is computationally extremely challenging (actually intractable) even for small number of sites, and approximate algorithms must be utilized (Harris et al., 2017). Harris et al. (2017) developed an efficient Bayesian fitting framework by approximating the neutral models with the hierarchical Dirichlet process (HDP). The approximation was able to encapsulate the three essential elements of Hubbell (2001) UNTB, as stated above, but offers an efficient Bayesian fitting strategy for the multi-site UNTB.

Sloan et al. (2006, 2007) showed that for large local population sizes, assuming a fixed finite-dimensional metacommunity distribution with *S* species present, the local community distribution, $\bar{\pi }$, can be approximated by a *Dirichlet* distribution (Sloan et al., 2006, 2007). But it was Harris et al. (2017) who developed the general framework for approximating the UNTB computationally efficiently. Assuming there is a potentially infinite number of species that can be observed in the local community, then the stationary distribution of observing local population *i* is a Dirichlet process (DP), i.e.,

(2) $$\bar{\pi_{i}}|I_{i},\bar{\beta }∼D\,P\,\left(I_{i},\bar{\beta }\right)$$

where $\bar{\beta }=\left(\beta_{1},\ldots ,\beta_{S}\right)$ is the relative frequency of each species in the metacommunity, and *I*_*i*_ is the immigration rate.

At the metacommunity level, a Dirichlet process can still be used, but the base distribution is simply a uniform distribution over arbitrary species labels. The metacommunity distribution is then a purely stick breaking process, i.e.,

(3) $$\bar{\beta }∼S\,t\,i\,c\,k\,\left(\theta \right)$$

where θ is the fundamental biodiversity number. θ is a function of *speciation rate* (*s*) in the form of θ = (*s*/(1−*s*)(*N*−1), where *N* is the size of metacommunity (i.e., the fixed number of individuals in the metacommunity). The total number of species (*S*) in the metacommunity proportionally increases with θ. In addition, when θ increases, the SAD (species abundance distribution) is increasingly skewed to low abundance rare species (Harris et al., 2017). Note that speciation in the metacommunity is a counterpart of migration in a local community, except that the speciation is in operation on a longer timescale than migration. For this reason, both immigration rate (*I*_*i*_) and biodiversity number θ have similar structure in their models. Specifically, *I*_*i*_ = (*m*_*i*_/(1−*m*_*i*_)(*N*_*i*_−1), where *m*_*i*_ is the immigration probability to local community *i*, and *N*_*i*_ is the local community size. Obviously, when *I*_*i*_→∝, the stationary distribution of local community should approach the metacommunity distribution since that means migration probability is equal to *1*, i.e., all members in the local community are immigrants. When *I*_*i*_→ 0, local community can become dominated by a single species (Harris et al., 2017).

Given that both local community and metacommunity are approximated with Dirichlet processes, the problem can be formulated as a hierarchical Dirichlet process (HDP) (Teh et al., 2006; Harris et al., 2017). Alternatively, Dirichlet process (DP) can also be formulated as the so-called Chinese restaurant process, from which the Antoniak equation can be derived. The Antoniak equation represents the number species (*S*) observed following *N* draws from a Dirichlet process with biodiversity number θ, and is with the following form:

(4) $$P\,\left(S|\theta ,N\right)=s\,\left(N,S\right)\,\theta^{S}\,\frac{\Gamma \,\left(\theta \right)}{\Gamma \,\left(\theta +N\right)}$$

where *s*(*N*, *S*) is the unsigned Stirling number of the first kind and Γ(.) denotes the gamma function (Antoniak, 1974).

#### Gibbs Sampler (MCMC Algorithm) for the UNTB-HDP Model

The full UNTB-HDP model is obtained by combining previous equations (1–3) and also the distribution models of biodiversity number (θ) and immigration rate (*I*_*i*_), both of which are assumed to follow Gamma distribution. Harris et al. (2017) developed an efficient Gibbs sampler for the UNTB-HDP approximation, which is a type of Bayesian Markov Chain Monte Carlo (MCMC) algorithm and can be summarized as the following four sampling steps, including sampling the biodiversity parameter, sampling the metacommunity distribution, sampling the immigration rate, and sampling the ancestral states. Harris et al. (2017) found through experiments that to ensure sampling was performed with the stationary distribution, 50,000 Gibb samples for each fitted dataset were necessary with the first 25,000 iterations removed as burn-in. The results are reported as the *median* values over the last 25,000 samples with upper and lower credible limits (Bayesian confidence) given by 2.5 and 97.5% quantiles of those samples.

#### Fitness Tests for the UNTB-HDP Multi-Site Neutral (MSN) Model

To determine whether an observed dataset fits the UNTB-HDP multi-site neutral (MSN) model (hereafter shortened as MSN model), Harris et al. (2017) proposed a similar Monte Carlo significance test to that used by Etienne (2007). Furthermore, Harris et al. (2017) also developed a procedure to test for the local neutral community assembly but with a fitted possibly non-neutral metacommunity because of the hierarchical nature of the MSN model. Specifically, with Harris et al. (2017) MSN model, two-level tests (local community and metacommunity levels) for neutrality can be performed. For both the tests, samples were generated from *N* = 2,500 sets of fitted MSN parameters, which were selected from every tenth iteration of the last 25,000 Gibbs samples (a total of 50,000 samples were simulated, and the first 25,000 samples were discarded as burn-in). *N* = 2,500 is chosen to compute the pseudo *P*-values for conducting the neutrality test (Harris et al., 2017). In addition, for each observed community sample, there is the *actual log-likelihood L*_0_. Two additional parameters θ and *M* are particular worthy of mentioning: θ is the *median* of the *fundamental biodiversity par*ameters computed from 25,000 times of simulations, and *M*-value is the average of the medians of the *migration rates* of local communities in each metacommunity, also computed from 25,000 times of simulations.

To test the neutrality at the metacommunity level, *P*_*M*_, which is “the proportion of the simulated neutral samples with their likelihoods *not* exceeding the observed data likelihood” (Harris et al., 2017). The computation of *P*_*M*_ is as follows: Assume *L*_*M*_ is the median of the log-likelihoods of the simulated neutral metacommunity samples, and *N*_*M*_ is the number of simulated neutral metacommunity samples, having their log-likelihoods satisfying *L* ≤ *L*_0_ (where *L* is the simulated likelihood and *L*_0_ is the actual likelihood as mentioned previously), then the *P*_*M*_ = *N_*M/*_N* is a pseudo *P*-value for testing the neutrality at metacommunity level. If *P*_*M*_ > 0.05, the metacommunity appears to satisfy the MSN model, according to Harris et al. (2017).

To test the neutrality at the local community level, *P*_*L*_, which is the proportion of the simulated locally neutral samples exceeding the observed data likelihood (Harris et al., 2017). It is computed as follows:

Assume *L*_*L*_ is the median of the log-likelihoods of the simulated local community samples, and *N*_*L*_ is the number of simulated local community samples, having their likelihoods not exceeding the *L*_0_, then *P_L_* = *N_*L*__/_N*, is the pseudo *P*-value for testing the neutrality at the local community level. If *P*_*L*_ > 0.05, the local community appears to satisfy the neutral model. Readers are referred to Harris et al. (2017) for the detailed algorithm and computational procedures (including the software in C language) for fitting the MSN model, which we used for analyzing HVM datasets in this study.

### The Niche-Neutral Hybrid (NNH) Model by Tang and Zhou (2013)

Tang and Zhou (2013) proposed a hybrid niche-neutral model by revising Volkov et al. (2007) neutral model for multiple discrete communities. Volkov et al. (2007) assumed that the inter-species interactions in a steady-state community may be ignored, and all species in the community become functionally equivalent. They further assumed that birth and death probabilities of a species with *n* individuals are *b*_*n*_ = *b*(*n* + γ) and *d*_*n*_ = *dn*, respectively, where *b* and *d* are the per-capita density-independent birth and death rates, and γ is a parameter for immigration. The migration was assumed to be species-independent, corresponding to immigration from a time-averaged metacommunity in a species-symmetric manner. This treatment of migration, in effect, ignored any immigration between local communities within the metacommunity, and also, the rates of immigration considered were small. By solving the master equation for the dynamics of a species, Volkov et al. (2007) obtained the probability that a species has *n* individuals, which follows the negative binomial distribution:

(5) $$p\,\left(n\right)=\frac{{\left(1-x\right)}^{\gamma }}{\Gamma \,\left(\gamma \right)}\,\frac{x^{n}}{n!}\,\Gamma \,\left(n+\gamma \right)$$

where *x* is the ratio of the *per* capita birth to death rate (i.e., *b*/*d*, a measure of the lifetime reproductive success), and $\Gamma \,\left(z\right)=\int_{0}^{\infty }t^{z-1}\,e^{-t}\,dt$, which is equal to (*z*-1)! for integer *z*. They further obtained the mean number of species with abundance *n*:

(6) $$<\varphi_{n}\geq \theta \,\frac{x^{n}}{n!}\,\Gamma \,\left(n+\gamma \right)$$

where θ is the fundamental biodiversity parameter, and *S* is the number of observed species.

Tang and Zhou assumed that a semi-isolated local community consists of *K* non-overlapping niches. Within each niche, a number of species follow their own neutral rules independent of the other *K*-1 niches. By applying Volkov et al. (2007) neutral model for multiple discrete communities to a single niche of the community, Tang and Zhou (2013) derived the expected number of species with abundance *n* in niche *i* as:

(7) $$<\varphi_{n,i}\geq \theta_{i}\,\frac{x_{i}^{n}}{n!}\,\Gamma \,\left(n+\gamma_{i}\right)$$

where *θ_*i*_* is the biodiversity parameter for niche *i*, *x*_*i*_ is the ratio of per capita birth to death rates of each species in niche *i*, and γ*_*i*_* is a parameters for immigration of niche *i*. The total expected number of species with abundance *n* in the community consisting of *K* niches is represented by the following equation:

(8) $$<\varphi_{n};K\geq \sum_{i=1}^{K}<\varphi_{n,i}>$$

Note that Eq. 8 is a summation of Eq. 7 across *K* niches, i.e., summing up all species with an abundance of *n* across all *K* niches. The following Chi-squared test statistic is utilized to determine the goodness-of-fitting for the niche-neutral hybrid model, i.e.,

(9) $$\chi^{2}=\sum_{n}\frac{{\left(E_{n}-O_{n}\right)}^{2}}{E_{n}}$$

where *E*_*n*_ is the expected number of species with abundance *n*, *O*_*n*_ is the observed number of species with abundance *n*.

To test the niche-neutral hybrid effects with Tang and Zhou (2013) NNH model, we computed the following items (listed in Supplementary Table 2 of the online supplementary information (OSI) and partially in Table 3), including: the average number of individuals per niche (local community) in each metacommunity (*J*), the average species numbers per niche (local community) in each metacommunity (*S*), the average fundamental biodiversity parameter per niche (local community) in each metacommunity (θ), the average of the migration coefficients (*m*), the average of the birth to death ratio (*x*), the average of the migration rate (γ). To conduct the χ^2^-test at the meta-community level, we computed χ^2^-value [Eq. 9] and associated *P*-value. To test the neutrality at a local community level, Volkov et al. (2003, 2007) approach for fitting the relative species abundance (RSA) distribution to their neutral model is adopted. Specifically, we computed and reported (see the last two columns in Supplementary Table 2 and Table 3) the number and percentage of local communities (niches) that passed the local neutrality test.

The *P*-value of the Chi-squared test is then used to determine whether or not Tang and Zhou (2013) hybrid model is suitable for a series of microbial communities sampled from each individual. In the case of our time-series microbiome datasets, we treat each time point as a niche occupied by a local microbial community and fit the neutral model for each local community. Specifically, at the metacommunity level, if *P*-value > 0.05, then the metacommunity appears to satisfy the NNH, and the metacommunity assembly is co-driven by both niche and neutral processes, which also implies that the metacommunity itself does not satisfy the neutral theory, but within each niche, the local community is neutral. If *P*-value < 0.05, the metacommunity does not seem to satisfy the NNH, which also implies that within each niche, the local community is not neutral either, and the metacommunity assembly is solely influenced by the niche process. Readers are referred to Tang and Zhou (2013) for the detailed algorithm and computational procedures (including the software) for fitting the NNH model, which we used for analyzing HVM datasets in this study.

### Model Specificity and *P*-Value Threshold Setting in Testing the Null Models

Two uncertainties have been well recognized in testing the neutral theory and niche-neutral hybrid models including the previous MSN and NNH models. One is the lack of full model specificity in fitting the neutral and/or niche-neutral hybrid models such as MSN/HHH models, and another is the lack of definite rules for setting the *P*-value thresholds in testing null models. What makes the statistical inferences more difficult is the potential interdependence between both uncertainties. There are no silver bullets to resolve them for various reasons including the complexity of the problem *per se* and limitations of the *P*-value setting in frequentist approaches to statistical inferences. In this article, no perfect solutions are offered, but we present two measures to relieve both issues. First, to evaluate the specificity of the MSN/NNH models, we classify the model-fittings as four possible categories: MSN-only, NNH only, both MSN & NNH, neither MSN nor MSN, and further observe the change of category proportions when *P*-value thresholds were specified differently. This allows us, at the minimum, to have an educated guess for the specificity of each model, particularly under different confidence levels (*P*-values). Second, besides testing the null models (MSN/NNH) under the traditional “default” *P*-value = 0.05, the results of model testing under a series of *P*-value thresholds are presented and analyzed. The variable *P*-value thresholds allow us to assess the goodness-of-fitting of the MSN/NNH models under various levels of confidence.

## Results

### The Niche-Neutral Continuum in Shaping the HVM Dynamics Evaluated Under Traditional “Default” *P*-Value Threshold

**FIGURE 2:**
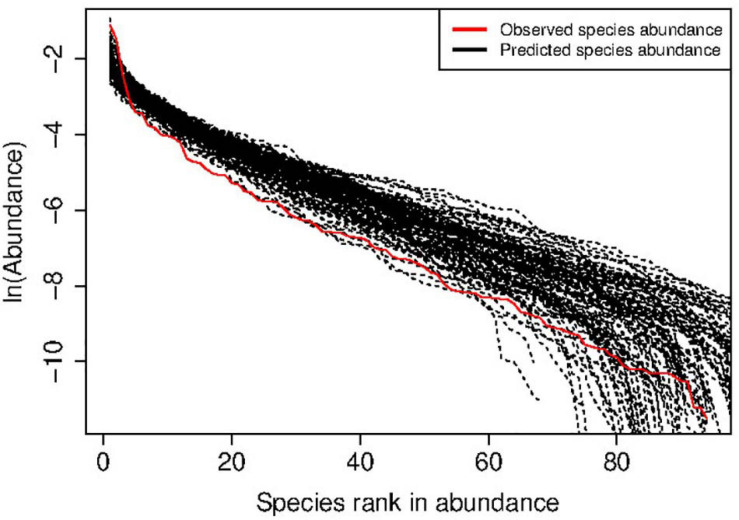
An example of human vaginal microbial metacommunity (Subject#s35: disease status = SBV) showing successful fitting to the MSN (multi-site neutral) model: the observed *species (relative) abundance* is estimated from 68 sampling times of the HVM of the subject, and the predicted species abundance is from 25,000 times of MSN simulations.

**FIGURE 3:**
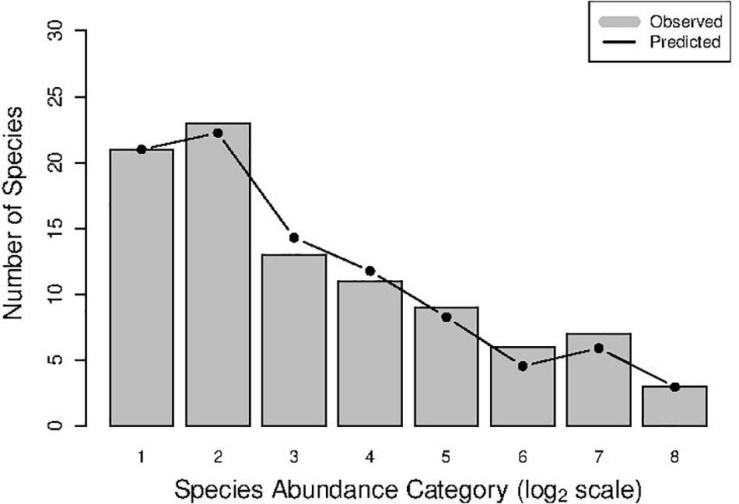
An example of human vaginal microbial metacommunity (Subject#s3: disease status = SBV) showing successful fitting to the NNH (neutral-niche hybrid) model: the *observed number of species* is estimated from approximately 59 sampling times of the HVM of the subject, and the predicted species number is from the NNH simulations.

Supplementary Table 1 in the OSI listed the *full* test results for the MSN with five HVM (human vaginal microbiome) datasets (groups) outlined in Table 1. Table 2 below was excerpted from Supplementary Table 1 to exhibit the results of 9 selected meta-communities. Similarly, Table 3 below exhibited the results of 9 selected meta-communities from Supplementary Table 2 in the OSI, where the full results for fitting the NNH model were listed. Figures 2, 3 illustrated two examples of fitting the MSN and NNH, respectively.

**TABLE 2:** Fitting the HDP-MSN (hierarchical Dirichlet process approximated multi-site neutral model) (Harris et al., 2017) to the HVM (human vaginal microbiome) datasets for selected individuals, excerpted from Supplementary Table 1 in the OSI*.

Datasets	Case No.	L_0_	θ	M-Value	Meta-Community				Local Community			
						
					L_*M*_	N_*M*_	N	P_*M*_	L_*L*_	N_*L*_	N	P_*L*_
ABV	S12	–7164.578	17.818	11.849	–8855.097	2437	2500	0.975	–7387.665	2158	2500	0.863
SBV	S5	–5929.666	15.552	7.295	–7861.124	2469	2500	0.988	–6220.505	2339	2500	0.936
	S3	–1749.538	4.759	5.645	–1945.254	1715	2500	0.686	–1781.817	1672	2500	0.669
	S17	–10498.771	8.166	920.730	–6555.105	0	2500	0.000	–2900.912	0	2500	0.000
Healthy-1	S7	–7330.538	12.554	17.957	–10193.354	2475	2500	0.990	–7530.997	2096	2500	0.838
Healthy-2	#400	–1609.574	12.332	9.409	–3248.037	2500	2500	1.000	–1766.803	2330	2500	0.932
	#401	–3002.396	23.955	6.088	–4053.368	2495	2500	0.998	–3362.560	2492	2500	0.997
Pregnancy	N002	–325.419	12.392	2.623	–447.710	2384	2500	0.954	–403.615	2378	2500	0.951
	N003	–581.146	13.098	7.923	–687.729	2099	2500	0.840	–648.312	2244	2500	0.898

*N = 2,500 is the number of Gibb samples selected from 25,000 simulated communities (*i.e.*, every tenth iteration of the last 25,000 Gibbs samples, a total of 50,000 simulations were performed and with the first 25,000 discarded as burn-in), and the *N* is used to compute the pseudo *P*-value below for conducting the neutrality test. *L*_0_ is the *actual (observed) log-likelihood*. θ is the median of biodiversity parameters computed from 25,000 times of simulations. *M*-value is the average medians of the migration rates of local communities in each metacommunity, also computed from 25,000 times of simulations. *L*_*M*_ is the median of the log-likelihoods of the simulated neutral metacommunity samples; and *N*_*M*_ is the number of simulated neutral metacommunity samples with their likelihoods satisfying the *L* < *L*_0_ (*L* is the simulated likelihood and *L*_0_ is the actually observed likelihood), *P*_*M*_ = *N_*M/*_N* is the pseudo *P*-value for testing the neutrality at metacommunity level; if *P*_*M*_ > 0.05, the metacommunity is indistinguishable from the prediction of the MSN model. *L*_*L*_ is the median of the log-likelihoods of the simulated local community samples, and *N*_*L*_ is the number of simulated local community samples with their likelihoods not exceeding the *L*_0_. *P*_*L*_ = *N_*L/*_N*, is the pseudo *P*-value for testing the neutrality at the local community level; if *P*_*L*_ > 0.05, the local community is indistinguishable from the neutral model. See Figure 2 for an example of successfully fitting to the MSN model.

**TABLE 3:** Fitting the NNH (niche-neutral hybrid) model (Tang and Zhou, 2013) to the HVM (human vaginal microbiome) datasets for selected individuals, excerpted from Supplementary Table 2 in the OSI*.

Datasets	ID	*J*	*S*	θ	*m*	*x*	γ	*R* ^2^	χ ^2^	*P*-value	*N* ^*pass*^	%*^(pass)^*
ABV	S12	5261.723	23.043	9277.776	0.000	0.691	0.488	0.996	52.253	0.000	22	46.8
SBV	S5	4647.896	18.042	466.265	0.001	0.643	0.557	0.983	154.291	0.000	18	37.5
	S3	164.000	8.455	4.032	0.007	0.803	1.236	0.981	0.925	0.996	11	100.0
	S17	5852.400	23.940	571.207	0.000	0.724	0.531	0.986	74.385	0.000	33	66.0
Healthy-1	S7	5902.193	20.421	650.047	0.000	0.687	0.462	0.986	87.923	0.000	13	37.1
Healthy-2	#400	2737.111	14.444	6.379	0.001	0.688	1.730	0.944	23.091	0.027	3	33.3
	#401	2614.200	22.933	7.292	0.000	0.760	1.242	0.983	12.745	0.310	13	86.7
Pregnancy	N002	4278.000	14.500	24.256	0.000	0.541	0.890	0.974	209327	0.000	1	50.0
	N003	4215.500	22.500	3.432	0.000	0.762	1.973	0.851	5.358	0.913	4	100.0

*J: the average number of individuals per niche (local community) in each metacommunity, *S*: the average species numbers per niche (local community) in each metacommunity, θ: the average fundamental biodiversity parameter per niche (local community) in each metacommunity, *m*: the average of the migration coefficients, *x*: the average of the birth to death ratio, γ: the average of the migration rate, *R*^2^: the goodness-of-fitting index, χ^2^-value: the χ^2^-value of chi-squared test for observed value against predicted value, *P*-value for the χ^2^-test; when *P*-value > 0.05, the metacommunity satisfies the NNH model. The last two columns are the number and percentage of local communities (niches) that passed the local neutrality test. Note that *R*^2^ = *1* resulted from approximation with four effective digits only (*e.g*., 0.99995, exact 1 is nearly impossible to achieve). See Figure 3 for an example of successfully fitting to the NNH model. Note that we use the *P*-value (FDR) after the FDR control was imposed to determine the outcome of testing the NNH model.

To better illustrate the full results in Supplementary Tables 1, 2 with Tables 2, 3, we selected 4 meta-communities from each of the five HVM datasets, corresponding to the 4 possible outcomes of testing the MSN and NNH simultaneously (i.e., passing MSN or NNH alone, passing both or passing neither). With this scheme, a maximal number of 20 (4×5) samples could be selected, and it turned out that 11 of the combinations were missing from the results, leading to only 9 meta-communities being selected in Tables 2, 3, respectively. The table legends were noted at the bottom sections of Tables 2, 3 below. Tables 2, 3, therefore, offer windows to inspect the parameters and infer findings from fitting the MSN/NNH models. To inspect the complete test results of the 79 meta-communities and 2,733 local communities, readers are referred to Supplementary Tables 1, 2 in the OSI.

**FIGURE 4:**
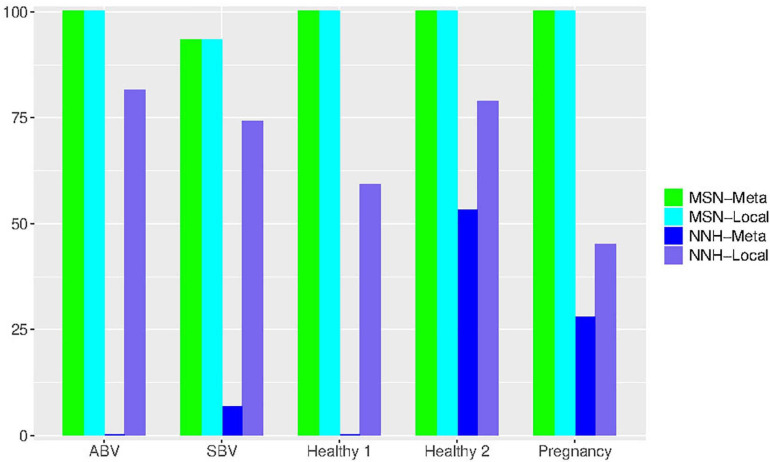
Bar chart showing the passing percentages of testing the MSN and NNH models (when *P*-value = 0.05) for each human vaginal microbiome dataset (group), respectively: for each group, the passing percentages for both meta- and local-community of each model (MSN or NNH) were exhibited.

We now try to draw a big picture from the test results (Supplementary Tables 1, 2 and Tables 2, 3) by computing the statistics of the passing rates from testing the MSN and NNH models. Recall that they use exactly the same data formats, i.e., with exactly the same specification for the local community and metacommunity. For example, with the dataset of “32-healthy” cohort, 32 subjects represented 32 meta-communities, and each metacommunity contained 25–33 local communities (or 25–33 *niches* in the case of NNH) given that each subject was sampled 25–33 times. Table 4 (also see Figure 4) below exhibited the passing rates for both MSN (the left) and NNH (the right) models; for each model, the passing rate at metacommunity level and local community level was listed separately. Note that in Table 4, the passing percentages for MSN/NNH corresponding to a series of *P*-value thresholds were presented, but here we only explain the result from the traditional “default” threshold (*P* = 0.05) and the results for other threshold values are explained in the following discussion section.

**TABLE 4:** The passing percentages for testing the MSN (multi-site neutral) and NNH (niche-neutral hybrid) models with the HVM datasets, summarized from Supplementary Tables 1, 2 and a series of the *P*-value thresholds (*P*_*t*_) for testing MSN/NNH were set to *P*_*t*_=0.05, 0.5, 0.9 or 0.95.

Microbiome	*N	Meta community				Local community			
			
		0.05	0.5	0.9	0.95	0.05	0.5	0.9	0.95
**The passing percentage (%) of MSN (Multi-site neutral) model**									
ABV	6	100	67.7	33.3	16.7	100	50	0	0
SBV	15	86.7	80	26.7	13.3	86.7	66.7	13.3	6.7
HEA-1	4	100	100	75	75	100	100	50	25
HEA-2	32	100	100	90.6	75	100	100	75	65.6
Pregnancy	22	100	100	86.4	68.2	100	100	31.8	13.6
Overall	79	97.3	89.5	62.4	49.6	97.3	83.3	34.0	22.2
**The passing percentage (%) of NNH (Niche-neutral hybrid) model**									
ABV	6	0	0	0	0	81.4	57.9	19.9	14.1
SBV	15	6.7	6.7	6.7	6.7	74.0	55.2	23.9	14.8
HEA-1	4	0	0	0	0	59.2	39.8	21.0	10.6
HEA-2	32	53.1	15.6	3.1	3.1	78.8	68.1	32.5	19.8
Pregnancy	18	27.8	16.7	16.7	5.6	44.9	35.6	22.2	9.3
Overall	75	17.52	7.8	5.3	3.08	67.6	51.3	23.9	13.7

*N is the number of meta-communities (the number of individual subjects) investigated in each dataset.

First, regarding the overall performance of the MSN model, 97.3% of meta-communities and local communities passed the neutrality test, respectively. The range of neutrality percentage was 86.7–100% across five datasets. Therefore, the stochastic neutral forces seem to play a dominant role in shaping the assembly of HVMs. At a local community level, the performance of NNH is significantly lower than that of MSN, with local neutrality passing the neutrality test at rate of 67.6%. However, at the metacommunity level, NNH also exhibited a moderate 17.5% of passing rate. Overall, niche differentiations appear to be moderate in the HVMs. In summary, the above findings indicate that both neutral and niche forces are in effect in shaping the community dynamics in the HVMs, but the neutral effects seem to play a dominant role.

### The Effects of BV on the Neutral-Niche Continuum in the HVM

We further investigated the influence of BV (bacterial vaginosis) including both SBV (symptomatic BV) and ABV (asymptomatic BV) on the balance between neutral and niche forces in shaping the HVM dynamics by performing Fisher’s exact test and Student’s *t*-test. The Fisher’s exact test was performed to evaluate the effect of BV on the rate of passing the neutrality test (MSN) or testing the niche-neutral hybrid effect (NNH) at the metacommunity level (the left side in Table 5), and Student’s *t*-test on the passing rate of neutrality test at the local community level with either MSN or NNH model (the right side in Table 5). Similar to the previous sub-section, here we only analyze the BV effects under traditional *P*-value = 0.05 threshold and delay the analyses under alternative *P*-value thresholds to the discussion section.

**TABLE 5:** The *P*-values from testing the difference between various groups (ABV, SBV, HEA-1, HEA-2, and HEA) in their passing rates (from testing the MSN/NNH models) with *Fisher exact test* for the meta-community or *Student*’*s t*-test for the local community (^#,^∗^^).

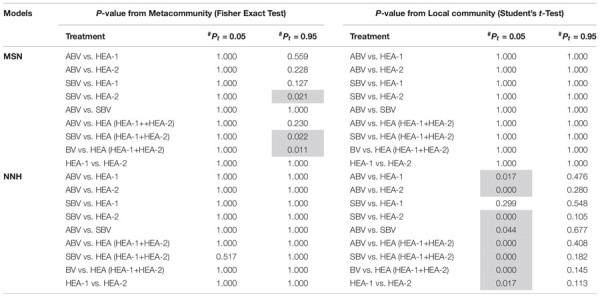

^#^The *P*-value thresholds for testing the MSN/NNH models were set to 0.05 and 0.95, corresponding to the two column heads “*P* = 0.05” and “*P* = 0.95”; be noted that they are column heads and are totally different the *P*-value*s* in the table entries that are from Fisher or *t*-tests. *The HEA1 group has 4 subjects only, and we suggest following the inferences from the HEA2 (32 subjects) or HEA (=HEA1+HEA2) in case there were conflicting results between HEA1 with the other groups. Shaded entries are comparisons with significant differences (*P* ≤ 0.05).

Interestingly, both MSN and NNH exhibited slightly different results regarding the effects of BV status on the passing percentages of model tests. At the local community level, there appears to be significant differences in BV (SBV) and HEA (healthy groups) (*P*-value < 0.05, Table 5) according to the NNH model. However, at the metacommunity level, regardless of the MSN or NNH, the differences between various groups were statistically insignificant. The lack of difference between the HEA and pregnancy groups is also expected. Romero et al. (2014) defined a normal pregnancy as a woman with no obstetrical, medical or surgical complications, and delivered at term (38 to 42 weeks) without complications. The pregnancy group studied by Romero consisted of 22 normal pregnancies. Therefore, it appears that no statistically significant differences were detected between various groups in terms of MSN or NNH testing except for NNH at local community scale.

## Discussion

With traditional neutral theory of biodiversity, the spatially explicit or implicit model describing the metacommunity consisting of multiple local communities is the most frequently used metacommunity model for testing neutrality (Hubbell, 2001; Rosindell et al., 2011, 2012; Ma, 2020). The use of longitudinal community/meta-community samples to perform dynamic analysis of the niches vs. neutrality and to further assess and interpret the community static and dynamic properties by harnessing the neutral theory has been few but can be equally effective (Kalyuzhny et al., 2014a, b, 2015). The integrated modeling with MSN/NNH in previous sections demonstrated another approach to generalizing the neutral-theoretic analysis to temporal meta-communities. Furthermore, we take advantages of a recent advance in computational statistics made by Harris et al. (2017) HDP-MSN machine learning algorithm. The HDP-MSN overcomes a significant computational bottleneck that existed in estimating the migration rates (*m*) when the number of local communities is large, which prevented large-scale testing of the UNTB with truly multi-site datasets. Nevertheless, truly multi-site datasets are scarce, especially in the studies of the human microbiome, where community samples are usually taken from unrelated individuals, and therefore dispersal (migration) among individuals is unlikely to occur on ecological timescales. In this study, we use the time-series sampling data in place of spatial sampling data. That is, the vaginal microbial community of each subject was sampled in patients at varying numbers of time points (6–60, see Table 1). By using time-series data with the MSN and NNH models, one can effectively evaluate the levels of stochastic *neutral* forces and deterministic *niche* forces in driving the community dynamics. In the case of time-series data, stochastic neutral forces may include stochastic fluctuations in demography (in the birth-death processes of bacterial cell divisions and deaths), which is the analog to ecological drift in neutral theory. The deterministic forces in time series data can include diversity- or dominance-dependent regulatory forces for community stability (dynamics) (Ma and Ellison, 2018, 2019).

As explained in the previous section of results, the results from the integrated niche-neutral hybrid analyses under a default *P*-value = 0.05 with MSN and NNH models in this study seem to suggest that neutral drifts play a dominant role in driving the community dynamics of the HVM, while the deterministic niche differentiation is moderate (approximately 17% in terms of passing NNH test). As further elaborated below, the assessment of the relative significance of neutral vs. niche may be strongly influenced by the model-choice (MSN or NNH) and *P*-value thresholds. It should also be reiterated that the conclusions obtained from this study are from analyzing the temporal dynamics data of the HVM, rather than from analyzing spatial metacommunity samples as usually performed in community ecology. In other words, the local communities in our analyses are simply the snapshots of an individual woman’s vaginal microbiome dynamics. Therefore, niche differentiations are also “temporal differentiations,” which might be relatively weak due to the nature of longitudinal observations. Future studies with “orthodox” spatial metacommunity samples should shed a more comprehensive picture on the community assembly and diversity maintenance of the HVMs. In the remainder of this article, we discuss two uncertainties regarding the test of the neutral and/or niche-neutral hybrid models.

First, it is well known that a significant challenge in investigating the mechanisms of community assembly or distinguishing the neutral from niche effects is that multiple independents models with possibly different ecological assumptions about the mechanisms may produce similar goodness-of-fittings to the same datasets, which is termed the lack of full model specificity in previous sections. This can make the inferences of definite mechanisms from different models difficult since the mapping from assumptions to mechanisms may not be one-to-one. Table 6 listed the breakups of successful fittings of the MSN/NNH models with *P*-value thresholds of 0.05–0.95, classified as four groups including successfully fitted to “MSN-only,” “NNH-only,” “both MSN & NNH,” and “neither MSN nor NNH.” Here, we first discuss the breakups when *P*-value threshold for testing the MSN/NNH model is set to 0.05, and the other thresholds are discussed shortly below. As exhibited in Table 6, overall, the “MSN-only group” (fitted to MSN uniquely) takes about 68% (ranged between 47 and 100%) of all cases and “both MSN & NNH” group (non-unique fittings) takes about 29%, (ranged between 0 and 53%) and in less than 3% cases (2 out of 79 individuals) neither MSN nor NNH model was fitted successfully. Therefore, in the majority (68% or 54 out of 79 individuals), the MSN model was able to uniquely interpret the neutral dynamics of the HVMs, when the *P*-value threshold for testing MSN/NNH was set to 0.05.

**TABLE 6:** Comparative summary of the performances of MSN and NNH models fitted to the human vaginal microbiome datasets of 79 subjects (meta-communities), summarized from Supplementary Tables 1, 2, under different *P*_*t*_-value thresholds for testing MSN/MMH models.

Microbiome	Meta-Community	MSN only		NNH only		Both MSN & NNH		NOT (MSN, NNH)	
					
		*N*	%	*N*	%	*N*	%	*N*	%
***P*_*t*_ = 0.05**									
ABV	6	6	100.0	0	0.0	0	0.0	0	0.0
SBV	15	12	80.0	0	0.0	1	6.7	2	13.3
HEA-1	4	4	100.0	0	0.0	0	0.0	0	0.0
HEA-2 (32-Cohort)	32	15	46.9	0	0.0	17	53.1	0	0.0
Pregnancy	22	17	77.3	0	0.0	5	22.7	0	0.0
Overall	79	54	68.4	0	0.0	23	29.1	2	2.5
***P*_*t*_ = 0.5**									
ABV	6	4	66.7	0	0.0	0	0.0	2	33.3
SBV	15	11	73.3	0	0.0	1	6.7	3	20.0
HEA-1	4	4	100.0	0	0.0	0	0.0	0	0.0
HEA-2 (32-Cohort)	32	27	84.4	0	0.0	5	15.6	0	0.0
Pregnancy	22	19	86.4	0	0.0	3	13.6	0	0.0
Overall	79	65	82.3	0	0.0	9	11.4	5	6.3
***P*_*t*_ = 0.9**									
ABV	6	2	33.3	0	0.0	0	0.0	4	66.7
SBV	15	4	26.7	1	6.7	0	0.0	10	66.7
HEA-1	4	3	75.0	0	0.0	0	0.0	1	25.0
HEA-2 (32-Cohort)	32	28	87.5	0	0.0	1	3.1	3	9.4
Pregnancy	22	17	77.3	1	4.5	2	9.1	2	9.1
Overall	79	54	68.4	2	2.5	3	3.8	20	25.3
***P*_*t*_ = 0.95**									
ABV	6	1	16.7	0	0.0	0	0.0	5	83.3
SBV	15	2	13.3	1	6.7	0	0.0	12	80.0
HEA-1	4	3	75.0	0	0.0	0	0.0	1	25.0
HEA-2 (32-Cohort)	32	24	75.0	1	3.1	0	0.0	7	21.9
Pregnancy	22	14	63.6	0	0.0	1	4.5	7	31.8
Overall	79	44	55.7	2	2.5	1	1.3	32	40.5

Second, another dilemma that may lead to uncertainty in testing the neutral or niche-neutral hybrid models is the choice of *P*-value threshold. Traditionally, the *P*-value was set to 0.05 in testing the neutral theory; when *P* > 0.05, the null hypothesis or model (satisfying the MSN or NNH model) cannot be rejected. In other words, when *P* > 0.05, the observed community is considered indistinguishable from what the theoretical model predicts. In previous sections, *P*-value = 0.05 was termed traditional “default.” However, one may set *P*-value to other threshold values. The higher the *P*-value is, the more likely (the higher likelihood) that the community is consistent with the model prediction. That is, when the *P*-value is set to higher threshold values, it is more difficult to reject the null model. In terms of the neutrality test based on the MSN model, it implies that accepting neutral hypothesis is more reliable (conservative). In terms of the NNH model, it implies that accepting non-neutrality (niche differentiation) is more reliable (conservative). Consequently, when larger *P*-value thresholds are adopted, the confidence (reliability) to accepting the null model (MSN or NNH) is raised and the confidence to reject the null model (MSM or NNH) is lowered. Table 4 listed the passing percentages (strictly, should be stated as percentage indistinguishable from model prediction) from testing MSN/NNH when *P*-value was set to 0.05, 0.5, 0.9, and 0.95, respectively. Obviously, as shown in Table 4, higher *P*-values correspond to a lower passing percentage of MSN-neutrality tests. When the *P*-value threshold was raised to 0.95, the passing percentage of MSN-neutrality test declined to approximately 50%, while the percentage was 97.3% when the *P*-value = 0.05. Raising the *P*-value threshold from 0.05 to 0.95 is a rather dramatic increase of the confidence level for not rejecting the null neutral model (or accepting the null model), still nearly half the metacommunities (microbiomes of individuals) passed the MSN model, suggesting that the neutral drifts indeed play a significant role in shaping the dynamics of the HVM.

**FIGURE 5:**
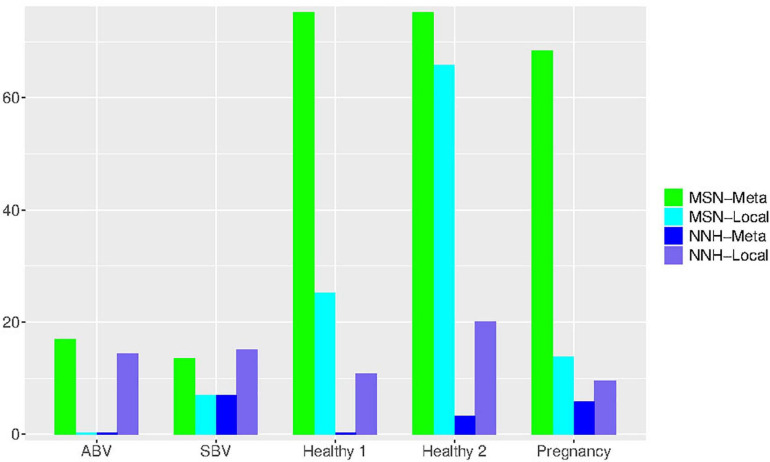
Bar chart showing the passing percentages of testing the MSN and NNH models (when *P*-value = 0.95) for each human vaginal microbiome dataset (group), respectively: for each group, the passing percentages for both meta- and local-community of each model (MSN or NNH) were exhibited.

An interesting observation is that, when the *P*-value threshold was set to default 0.05 (Table 4), the passing percentages of MSM testing were not significantly different between different treatments (see Table 5 for Fisher’s exact test). However, when the *P*-value threshold was set to 0.95 (Figure 5), the differences between the BV group (including ABV and SBV) and healthy groups (HEA1, HEA2, and pregnancy) were significant (13.3–16.7% vs. 68.2–75%). The healthy groups exhibited significantly more neutral communities than BV groups (Table 5). This result is actually puzzling. A traditional view has been that the vaginal microbiomes associated with BV usually have higher community diversity than the healthy counterparts, possibly due to the loss of dominant species such as *Lactobacillus* (e.g., Ma et al., 2012). Since communities with dominant species tend to contain more asymmetric interactions, they could be less likely neutral. Therefore, this puzzling result appears to contradict with the traditional view. Of course, the relationship between dominant species and non-neutrality is not necessarily positive; we hope that future more mechanistic studies will resolve the apparent inconsistency.

The adoption of different *P*-value thresholds may also affect the previously discussed specificity (uniqueness) of MSN/NNH model fittings. Table 6 shows the breakups of four categories (MSN-only, NNH-only, both MSN & NNH, neither MSN nor NNH) under different *P*-values ranging from *P*-value = 0.05 to 0.95. It appears that the model specificity of MSN seems to increase with the increase of *P*-value threshold adopted. This result should be expected since the increased *P*-value should raise the confidence level for accepting the null (MSN or NNH) model, and the passing percentages judged with higher confidence would decline accordingly.

Finally, the two previously discussed uncertainties associated with testing the neutral or niche-neutral hybrid models can be interdependent and proper resolving them requires both ecological science and statistical art. There may not be a perfect solution for resolving those issues due to both the ecological complexity and the limitation of frequentist statistical approaches. The art lies in balancing the trade-off between reliability (confidence) in hypothesis testing and model specificity. It is hoped that the demonstrative analysis and discussion included in this study with the HVMs will also be useful for other ecological and evolutionary modeling of biodiversity and biogeography.

## Data Availability Statement

The original contributions presented in the study are included in the article/Supplementary Material, further inquiries can be directed to the corresponding author.

## Author Contributions

ZM designed and performed the study and wrote the manuscript.

## Conflict of Interest

The author declares that the research was conducted in the absence of any commercial or financial relationships that could be construed as a potential conflict of interest.

## Publisher’s Note

All claims expressed in this article are solely those of the authors and do not necessarily represent those of their affiliated organizations, or those of the publisher, the editors and the reviewers. Any product that may be evaluated in this article, or claim that may be made by its manufacturer, is not guaranteed or endorsed by the publisher.

## Supplementary Material

Supplementary Table 1 Test results of fitting the MSN (multi-site neutral) model to the vaginal microbiome datasets.

Supplementary Table 2 Fitting the NNH (Niche-Neutral Hybrid) model to the vaginal microbiome datasets.
