# Supplementary material for: Evaluating the Assembly Dynamics in the Human Vaginal Microbiomes With Niche-Neutral Hybrid Modeling
Source: Front Microbiol. 2021 Aug 20;12:699939. doi: 10.3389/fmicb.2021.699939 (PMC8417885; doi:10.3389/fmicb.2021.699939)
Supplement: Supplementary Table 1 — Test results of fitting the MSN (multi-site neutral) model to the vaginal microbiome datasets. [file Table_1.pdf]

Supplementary Tables for: Ma ZS (2021) Evaluating the assembly dynamics in the human vaginal microbiomes with niche-neutral hybrid modeling. *Frontiers in Microbiology*

Table S1. Test results of fitting the MSN (multi-site neutral) model to the vaginal microbiome datasets*

ID	L_O	θ	*M-value*	*Metacommunity*					*Local community*				
				L_M	N_{MS}	N	$^{\#}P_{MS}$	P_M=1-P_M	L_L	N_{LS}	N	$^{\#}P_{LS}$	P_L=1-P_{LS}
**ABV**													
s12	-7164.578	17.818	11.849	-8855.097	63	2500	0.025	0.975	-7387.665	342	2500	0.137	0.863
s23	-15438.490	16.501	28.446	-13748.928	2186	2500	0.874	0.126	-15441.357	1235	2500	0.494	0.506
s27	-10366.906	11.368	46.280	-11729.422	480	2500	0.192	0.808	-10358.896	1301	2500	0.520	0.480
s60	-13585.931	15.981	25.854	-13386.112	1378	2500	0.551	0.449	-13559.163	1379	2500	0.552	0.448
s77	-9947.497	9.267	60.138	-13064.632	146	2500	0.058	0.942	-10049.400	690	2500	0.276	0.724
s82	-8210.227	8.086	26.506	-9174.771	664	2500	0.266	0.734	-7982.334	2303	2500	0.921	0.079
Passing rate with *P*-value=0.05								6(100%)					6 (100%)
**SBV**													
s3	-1749.538	4.759	5.645	-1945.254	785	2500	0.314	0.686	-1781.817	828	2500	0.331	0.669
s5	-5929.666	15.552	7.295	-7861.124	31	2500	0.012	0.988	-6220.505	161	2500	0.064	0.936
s15	-9037.927	14.276	14.980	-10775.122	202	2500	0.081	0.919	-9285.939	315	2500	0.126	0.874
s17	-10498.771	8.166	920.730	-6555.105	2500	2500	1.000	0.000	-2900.912	2500	2500	1.000	0.000
s35	-11536.919	15.087	21.498	-11707.390	1115	2500	0.446	0.554	-11662.499	658	2500	0.263	0.737
s40	-3422.864	12.627	1.888	-4109.700	178	2500	0.071	0.929	-3609.404	334	2500	0.134	0.866
s53	-8069.549	10.662	17.355	-8737.495	709	2500	0.284	0.716	-8026.233	1512	2500	0.605	0.395
s55	-14629.930	13.539	43.819	-12167.276	2361	2500	0.944	0.056	-14551.823	1730	2500	0.692	0.308
s59	-6821.856	15.369	7.480	-7409.555	554	2500	0.222	0.778	-7390.386	0	2500	0.000	1.000
s112	-11301.889	10.515	86.598	-12400.789	664	2500	0.266	0.734	-11410.515	614	2500	0.246	0.754
s116	-10807.837	10.325	48.837	-10958.763	1163	2500	0.465	0.535	-10738.380	1694	2500	0.678	0.322
s127	-7469.105	14.635	9.935	-8632.670	289	2500	0.116	0.884	-7669.177	378	2500	0.151	0.849
s128	-6229.250	11.534	10.238	-8866.448	28	2500	0.011	0.989	-6366.217	597	2500	0.239	0.761
s130	-10778.879	7.802	948.096	-7153.134	2496	2500	0.998	0.002	-3046.270	2500	2500	1.000	0.000
s135	-11641.403	14.683	20.728	-12725.909	567	2500	0.227	0.773	-11774.562	679	2500	0.272	0.728
Passing rate with *P*-value=0.05								13 (86.7%)					13 (86.7%)
**Healthy 1**													
s7	-7330.538	12.554	17.957	-10193.354	25	2500	0.010	0.990	-7530.997	404	2500	0.162	0.838
s49	-2532.916	4.096	21.308	-5810.465	8	2500	0.003	0.997	-2662.432	320	2500	0.128	0.872
s52	-11100.017	18.299	18.709	-11579.530	834	2500	0.334	0.666	-11472.099	109	2500	0.044	0.956
s96	-7365.433	12.862	13.804	-9406.521	96	2500	0.038	0.962	-7614.210	215	2500	0.086	0.914
Passing rate with *P*-value=0.05								4(100%)					4(100%)
**Healthy 2**													
#400	-1609.574	12.332	9.409	-3248.037	0	2500	0.000	1.000	-1766.803	170	2500	0.068	0.932
#401	-3002.396	23.955	6.088	-4053.368	5	2500	0.002	0.998	-3362.560	8	2500	0.003	0.997
#402	-2441.396	22.366	5.317	-3613.167	0	2500	0.000	1.000	-2780.164	7	2500	0.003	0.997
#403	-1021.129	5.454	8.815	-2250.915	8	2498	0.003	0.997	-1160.862	117	2498	0.047	0.953
#404	-3654.522	19.925	10.471	-4440.641	98	2500	0.039	0.961	-4059.332	1	2500	0.000	1.000
#405	-928.028	8.161	19.371	-3380.831	0	2500	0.000	1.000	-1013.098	387	2500	0.155	0.845
#406	-3122.365	17.703	8.204	-4053.569	41	2500	0.016	0.984	-3460.927	12	2500	0.005	0.995
#407	-3540.619	29.241	8.407	-4635.937	1	2500	0.000	1.000	-4014.006	1	2500	0.000	1.000
#408	-2740.120	9.861	16.541	-3506.609	156	2500	0.062	0.938	-2892.575	84	2500	0.034	0.966
#410	-2658.255	12.437	25.654	-4649.908	2	2500	0.001	0.999	-2853.935	118	2500	0.047	0.953
#411	-3924.919	17.048	17.836	-4737.801	158	2500	0.063	0.937	-4143.379	75	2500	0.030	0.970
#412	-879.529	6.599	8.922	-2120.737	2	2500	0.001	0.999	-909.919	889	2500	0.356	0.644
#413	-1980.947	9.127	12.383	-2299.806	438	2500	0.175	0.825	-2061.894	441	2500	0.176	0.824
#414	-2551.459	8.630	31.866	-3458.055	120	2500	0.048	0.952	-2624.196	510	2500	0.204	0.796
#415	-1736.665	9.971	15.935	-3776.182	0	2500	0.000	1.000	-1884.706	197	2500	0.079	0.921
#416	-1818.537	11.400	24.058	-3404.067	2	2500	0.001	0.999	-1908.899	392	2500	0.157	0.843
#418	-1492.991	12.903	23.687	-3802.482	0	2500	0.000	1.000	-1648.957	148	2500	0.059	0.941

#420	-2137.878	16.568	3.880	-2719.721	60	2500	0.024	0.976	-2421.298	25	2500	0.010	0.990
#423	-2854.868	18.160	22.004	-6116.863	0	2500	0.000	1.000	-3272.123	2	2500	0.001	0.999
#424	-3748.916	24.235	26.657	-7041.874	0	2500	0.000	1.000	-4115.412	14	2500	0.006	0.994
#429	-2174.709	12.306	32.867	-5421.585	0	2500	0.000	1.000	-2491.781	15	2500	0.006	0.994
#430	-4626.373	13.746	49.231	-5662.362	177	2500	0.071	0.929	-4802.823	108	2500	0.043	0.957
#431	-2785.901	10.876	13.780	-3259.166	391	2500	0.156	0.844	-2971.551	99	2500	0.040	0.960
#432	-2453.004	23.049	5.457	-3691.421	0	2500	0.000	1.000	-2882.853	0	2500	0.000	1.000
#435	-2176.750	11.146	24.253	-3400.387	16	2500	0.006	0.994	-2298.862	253	2500	0.101	0.899
#436	-9362.877	33.048	68.908	-10029.317	553	2500	0.221	0.779	-9797.443	5	2500	0.002	0.998
#437	-2053.210	17.762	19.624	-5142.038	0	2500	0.000	1.000	-2373.046	5	2500	0.002	0.998
#439	-2223.025	6.190	38.099	-3190.330	140	2500	0.056	0.944	-2276.686	635	2500	0.254	0.746
#443	-3280.197	29.978	6.143	-4091.676	17	2500	0.007	0.993	-3694.031	4	2500	0.002	0.998
#444	-5105.232	21.075	22.781	-6279.180	76	2500	0.030	0.970	-5394.874	27	2500	0.011	0.989
#445	-1818.573	7.410	9.018	-2514.747	150	2500	0.060	0.940	-1922.802	271	2500	0.108	0.892
#446	-1534.507	9.812	24.004	-3742.298	0	2500	0.000	1.000	-1739.310	35	2500	0.014	0.986
	Passing rate with P-value=0.05							32(100%)					32(100%)
						**Pregnancy**							
N001	-266.501	5.197	4.831	-390.001	220	2500	0.088	0.912	-302.183	440	2500	0.176	0.824
N002	-325.419	12.392	2.623	-447.710	116	2500	0.046	0.954	-403.615	122	2500	0.049	0.951
N003	-581.146	13.098	7.923	-687.729	401	2500	0.160	0.840	-648.312	256	2500	0.102	0.898
N004	-238.738	3.744	31.332	-561.912	24	2500	0.010	0.990	-270.517	470	2500	0.188	0.812
N005	-283.199	13.337	1.824	-409.214	99	2500	0.040	0.960	-364.458	138	2500	0.055	0.945
N006	-203.278	4.250	26.582	-403.059	43	2500	0.017	0.983	-215.829	852	2500	0.341	0.659
N007	-190.091	2.720	29.564	-440.169	50	2500	0.020	0.980	-216.558	509	2500	0.204	0.796
N008	-466.114	22.252	2.885	-580.535	177	2500	0.071	0.929	-561.103	113	2500	0.045	0.955
N009	-1116.626	12.300	19.482	-1325.947	340	2500	0.136	0.864	-1159.808	615	2500	0.246	0.754
N010	-288.383	9.661	4.831	-427.572	99	2500	0.040	0.960	-331.247	511	2500	0.204	0.796
N011	-310.569	13.235	1.516	-442.582	83	2500	0.033	0.967	-405.599	89	2500	0.036	0.964
N012	-146.112	3.241	24.926	-477.570	9	2500	0.004	0.996	-177.615	414	2500	0.166	0.834
N013	-348.614	7.036	34.753	-919.315	2	2500	0.001	0.999	-411.643	235	2500	0.094	0.906
N014	-233.858	10.846	9.630	-337.932	117	2500	0.047	0.953	-264.456	590	2500	0.236	0.764
N015	-431.536	6.332	18.017	-645.943	104	2500	0.042	0.958	-471.155	467	2500	0.187	0.813
N016	-105.893	2.151	21.030	-287.567	68	2500	0.027	0.973	-120.096	732	2500	0.293	0.707
N017	-238.013	3.461	22.517	-553.426	27	2500	0.011	0.989	-253.705	837	2500	0.335	0.665
N018	-423.815	5.679	11.518	-601.431	223	2500	0.089	0.911	-469.014	384	2500	0.154	0.846
N019	-356.798	10.868	2.244	-474.323	190	2500	0.076	0.924	-428.632	194	2500	0.078	0.922
N020	-204.344	6.938	3.626	-260.715	427	2500	0.171	0.829	-229.697	596	2500	0.238	0.762
N021	-611.249	21.759	2.873	-800.348	69	2500	0.028	0.972	-719.341	150	2500	0.060	0.940
N022	-331.183	8.065	12.118	-667.703	5	2500	0.002	0.998	-385.866	299	2500	0.120	0.880
	*Passing rate with P-value=0.05							22(100%)					22(100%)
	*Total Passing rate with P-value=0.05							78(98.7%)					78(98.7%)

*See Table 2 in the main text for the column legends and Material and Methods section for the detailed explanation. Due to the typo/error in Harris *et al.* (2017), the $\boldsymbol{P_M}$-values exhibited here should be adjusted as ($\boldsymbol{P_M}$=1−$\boldsymbol{P_{MS}}$), where $\boldsymbol{P_{MS}}$ is output from their computational program. Similarly, the $\boldsymbol{P_L}$-values should be adjusted as ($\boldsymbol{P_L}$=1−$\boldsymbol{P_{LS}}$), where $\boldsymbol{P_{LS}}$ is output from their computational program. Similarly, the $\boldsymbol{N_{MS}}$ and $\boldsymbol{N_{LS}}$ reported here are from the output of Harris et al. (2017) software. When computing the P-value used for testing the MSN-neutrality, their "complements" (N_M=2500−N_{MS}) or (N_L=2500−N_{LS}) should be used to circumvent their error.

** The passing rate here refers to the percentage of individuals with their microbiome species abundance distributions are *indistinguishable* from what are predicted by the MSN model at the P-value threshold was set to 0.05. As explained in the main manuscript, the P-value thresholds other than the tradition default P=0.05 may be adopted to perform the tests (*see* Tables 4 & 5).

Table S2. Fitting the NNH (Niche-Neutral Hybrid) model to the vaginal microbiome datasets*

Group ID	J	S	θ	m	x	γ	R^2	χ^2	P-value	N_{pass}	$\%_{(pass)}$
**ABV**											
S12	5261.723	23.043	9277.776	0.000	0.691	0.488	0.996	52.253	0.000	22	46.8
S23	3661.385	41.754	45.071	0.000	0.777	0.648	0.983	25.316	0.008	45	69.2
S27	3881.841	29.683	34.867	0.000	0.801	0.679	0.968	27.217	0.007	54	85.7
S60	5177.516	35.438	79.991	0.000	0.781	0.565	0.985	31.213	0.002	39	60.9
S77	7135.246	25.681	81.699	0.000	0.777	0.526	0.931	91.335	0.000	49	71.0
S82	7686.310	21.052	424.248	0.000	0.779	0.564	0.969	81.311	0.000	39	67.2
Passing rate with P-value=0.05									0	41.3	66.8
**SBV**											
S3	164.000	8.455	4.032	0.007	0.803	1.236	0.981	0.925	0.996	11	100.0
S5	4647.896	18.042	466.265	0.001	0.643	0.557	0.983	154.291	0.000	18	37.5
S15	5748.159	24.048	558.195	0.000	0.699	0.512	0.978	208.236	0.000	38	60.3
S17	5852.400	23.940	571.207	0.000	0.724	0.531	0.986	74.385	0.000	33	66.0
S35	4337.344	31.115	290.657	0.000	0.748	0.582	0.975	84.312	0.000	39	63.9
S40	6754.900	10.967	100.930	0.000	0.753	0.422	0.962	167.096	0.000	17	56.7
S53	7622.949	21.729	1143.323	0.000	0.731	0.461	0.976	68.198	0.000	36	61.0
S55	3242.484	42.032	189.097	0.000	0.786	0.776	0.954	63.784	0.000	25	40.3
S59	4131.681	20.574	293.489	0.000	0.621	0.502	0.990	132.587	0.000	11	23.4
S112	4926.635	34.413	52.072	0.000	0.761	0.584	0.974	80.014	0.000	28	44.4
S116	3644.619	32.603	38.783	0.000	0.800	0.625	0.991	22.330	0.022	53	84.1
S127	5859.623	19.792	2346.281	0.000	0.729	0.584	0.985	90.145	0.000	33	62.3
S128	6233.879	15.397	263.805	0.000	0.738	0.446	0.988	68.108	0.000	26	44.8
S130	5020.452	21.903	63.222	0.000	0.809	0.924	0.934	49.257	0.000	23	74.2
S135	6385.242	28.306	64738.113	0.000	0.772	0.535	0.979	30.309	0.003	36	58.1
Passing rate with P-value=0.05									1 (6.7%)	28.5	58.5
**Healthy 1 (HEA-1)**											
S7	5902.193	20.421	650.047	0.000	0.687	0.462	0.986	87.923	0.000	13	37.1
S49	8247.114	9.486	6921.418	0.000	0.655	0.318	0.978	173.072	0.000	12	20.0
S52	5343.267	31.217	777.053	0.000	0.655	0.578	0.989	145.420	0.000	20	35.1
S96	4047.655	20.982	216.799	0.000	0.723	0.575	0.954	81.246	0.000	21	38.2
Passing rate with P-value=0.05									0	16.5	32.6
**Healthy 2 (Vaginal 32) (HEA-2)**											
400	2737.111	14.444	6.379	0.001	0.688	1.730	0.944	23.091	0.027	3	33.3
401	2614.200	22.933	7.292	0.000	0.760	1.242	0.983	12.745	0.310	13	86.7
402	2145.583	20.833	7.752	0.000	0.807	0.935	0.980	5.982	0.817	11	91.7
403	2588.000	13.400	6.163	0.000	0.783	0.792	0.956	61.980	0.000	3	60.0
404	2376.667	26.429	8.236	0.000	0.773	1.136	0.974	15.205	0.173	17	81.0
405	2696.286	10.714	7.514	0.000	0.663	0.868	0.951	46.351	0.000	3	42.9
406	2973.222	23.889	9.321	0.000	0.746	1.079	0.968	18.237	0.109	7	77.8
407	2437.240	24.960	11.169	0.000	0.731	1.062	0.992	35.408	0.000	16	64.0
408	2431.444	18.556	3.555	0.001	0.732	1.830	0.821	8.176	0.612	8	88.9
410	2749.600	20.067	7.924	0.000	0.778	0.956	0.972	15.883	0.146	11	73.3
411	2499.933	30.000	7.763	0.001	0.773	1.280	0.980	19.507	0.053	14	93.3
412	2636.333	12.000	4.447	0.001	0.836	1.446	0.637	6.427	0.377	3	100.0
413	852.500	17.000	6.192	0.001	0.805	1.041	0.967	9.913	0.358	15	93.8
414	1136.444	19.389	4.977	0.001	0.789	1.294	0.953	6.981	0.639	18	100.0
415	2832.333	19.000	8.616	0.000	0.734	1.047	0.972	17.447	0.065	4	66.7
416	1393.923	17.077	6.674	0.001	0.803	0.896	0.924	23.704	0.014	11	84.6
418	1338.182	17.364	14.431	0.001	0.675	0.851	0.977	15.743	0.107	5	45.5
420	3393.462	15.000	5.499	0.001	0.740	1.360	0.961	60.507	0.000	10	76.9
423	5125.952	22.429	13.151	0.000	0.652	1.025	0.973	90.846	0.000	8	38.1
424	4098.909	27.682	11.950	0.000	0.716	1.045	0.983	55.693	0.000	13	59.1
429	4933.474	18.526	12.556	0.000	0.641	0.995	0.982	133.747	0.000	4	21.1
430	3866.667	34.583	7.915	0.000	0.797	1.213	0.887	15.493	0.115	12	100.0

431	2367.000	23.846	7.147	0.001	0.732	1.309	0.916	21.869	0.016	10	76.9
432	2953.706	20.176	11.642	0.000	0.682	0.990	0.949	35.194	0.000	7	41.2
435	2293.125	21.063	7.150	0.000	0.821	0.890	0.970	11.128	0.433	16	100.0
436	1995.517	70.241	28.131	0.000	0.749	0.929	0.980	34.670	0.000	21	72.4
437	2082.579	17.632	10.345	0.001	0.693	1.016	0.987	37.285	0.000	11	57.9
439	2298.417	15.333	2.176	0.001	0.788	1.959	0.952	1.760	0.995	12	100.0
443	3887.455	26.182	9.356	0.000	0.807	0.836	0.929	21.607	0.042	9	81.8
444	4327.600	35.100	7.430	0.000	0.775	1.383	0.960	11.889	0.372	19	95.0
445	1746.143	12.286	0.739	0.001	0.724	2.426	0.741	7.384	0.689	7	100.0
446	3855.846	17.615	10.316	0.000	0.737	1.314	0.980	27.306	0.007	10	76.9
Passing rate with *P*-value=0.05									17 (53.1%)	10.3	74.4
**Pregnancy**											
N002	4278.000	14.500	24.256	0.000	0.541	0.890	0.974	209327.	0.000	1	50.0
N003	4215.500	22.500	3.432	0.000	0.762	1.973	0.851	5.358	0.913	4	100.0
N005	4125.333	11.333	6.675	0.000	0.764	0.763	0.771	30.332	0.000	0	0.0
N006	2040.000	11.000	3.757	0.000	0.971	0.510	0.812	0.484	0.993	1	100.0
N007	3833.000	5.000	0.196	0.001	0.735	2.934	-0.010	0.571	0.903	1	100.0
N008	3637.750	17.750	7.930	0.000	0.779	0.826	0.819	15.582	0.049	3	75.0
N009	4198.250	29.500	5.590	0.000	0.860	1.189	0.630	14.790	0.192	4	100.0
N010	4123.333	13.667	10.473	0.000	0.571	1.042	0.937	230.681	0.000	1	33.3
N011	4770.000	11.500	8.269	0.000	0.608	0.953	0.881	173.183	0.000	0	0.0
N012	4062.333	6.333	8.312	0.000	0.393	1.143	0.875	12107.4	0.000	0	0.0
N013	4719.667	14.667	9.093	0.000	0.651	0.937	0.800	62.214	0.000	0	0.0
N014	4491.000	15.000	25.709	0.000	0.336	1.084	0.875	45072.0	0.000	1	100.0
N015	3553.667	15.667	8.332	0.000	0.727	0.872	0.874	84793.4	0.000	2	66.7
N017	3130.000	9.000	7.157	0.000	0.543	1.029	0.826	197.394	0.000	1	100.0
N019	3975.333	10.333	8.247	0.000	0.498	1.677	0.932	561311.	0.000	1	33.3
N020	3021.000	9.500	17.687	0.001	0.438	1.820	0.881	7.450	0.384	1	50.0
N021	4022.000	15.500	9.624	0.000	0.680	0.885	0.920	33.682	0.000	0	0.0
N022	4087.000	14.500	13.301	0.000	0.455	1.187	0.912	4357.46	0.000	0	0.0
Passing rate with *P*-value=0.05									5 (27.8%)	1.2	50.4
Total Passing rate with *P*-value=0.05									23 (30.1%)		

*See Table 3 in the main text for the column legends and Material and Methods section for the detailed explanation.

** The passing rate here refers to the percentage of individuals with their microbiome species abundance distributions are *indistinguishable* from what are predicted by the NNH model at the *P*-value threshold was set to 0.05. As explained in the main manuscript, the *P*-value thresholds other than the tradition default *P*=0.05 may be adopted to perform the tests (*see* Tables 4 & 5).
